# Supplementary material for: Decorin Concentrations in Aqueous Humor of Patients with Diabetic Retinopathy
Source: Life (Basel). 2021 Dec 17;11(12):1421. doi: 10.3390/life11121421 (PMC8707400; doi:10.3390/life11121421)
Supplement: Supplementary file 1 [file life-11-01421-s001.zip › life-1471524-supplementary.pdf]

life

MDPI

Supplementary Material of Decorin Concentrations in Aqueous Humor of Patients with Diabetic Retinopathy

Table S1. Sample numbers for each individual group.

Sample Numbers	
**Group**	**Sample Size (*n*)**
Total	82
Controls (total)	26
DR (total)	56
Female Control	8
Female DR	13
Male Control	18
Male DR	43
ETDRS Class 1	3
ETDRS Class 2	13
ETDRS Class 3	9
ETDRS Class 4	31
Gloucestershire R1M0	11
Gloucestershire R2M0	8
Gloucestershire R3M0	9
Gloucestershire R3M1	28
Treatment Naïve (TN)	28
Treatment Responders (TRes)	9
Treatment Recurrent (TRec)	8
Treatment Non-Responders (TnR)	11

Table S2. Student's *t*-test comparing decorin concentrations in control and DR groups based on sex.

Student's *t*-Test		
**Comparison**	**Mean ± SE**	***P* value**
Female Control vs. Male Control		0.0769
Female Control (*n* = 8)	3.506 ± 0.2292	
Male Control (*n* = 18)	3.647 ± 0.1481	
Female DR vs. Male DR		0.0003
Female DR (*n* = 13)	3.657 ± 0.1530	
Male DR (*n* = 43)	3.851 ± 0.1847	
Female Control vs. Female DR		0.0575
Female Control (*n* = 8)	3.506 ± 0.2292	
Female DR (*n* = 13)	3.657 ± 0.1530	
Male Control vs. Male DR		0.0001
Male Control (*n* = 18)	3.647 ± 0.1481	
Male DR (*n* = 43)	3.851 ± 0.1847	

Table S3. Tukey's pairwise comparison between ETDRS groups.

Effect	Comparison	*P* value
ETDRS	Ctrl vs. 1	0.5467
ETDRS	Ctrl vs. 2	0.7083
ETDRS	Ctrl vs. 3	0.6747
ETDRS	Ctrl vs. 4	0.0009

Effect	Comparison	*P* value
ETDRS	1 vs. 2	0.9340
ETDRS	1 vs. 3	0.9694
ETDRS	1 vs. 4	0.9996
ETDRS	2 vs. 3	0.9997
ETDRS	2 vs. 4	0.3302
ETDRS	3 vs. 4	0.6077

Table S4. Tukey's pairwise comparison between Gloucestershire groups.

Effect	Comparison	*P* value
Gloucestershire	R0M0 vs. R1M1	0.8061
Gloucestershire	R0M0 vs. R2M1	0.5980
Gloucestershire	R0M0 vs. R3M0	0.0061
Gloucestershire	R0M0 vs. R3M1	0.0613
Gloucestershire	R1M1 vs. R2M1	0.9886
Gloucestershire	R1M1 vs. R3M0	0.1414
Gloucestershire	R1M1 vs. R3M1	0.7032
Gloucestershire	R2M1 vs. R3M0	0.4759
Gloucestershire	R2M1 vs. R3M1	0.9792
Gloucestershire	R3M0 vs. R3M1	0.6374

Table S5. Tukey's pairwise comparison between treatment response groups.

Effect	Comparison	*P* value
Response	Ctrl vs. TnR	0.0038
Response	Ctrl vs. TRec	0.0812
Response	Ctrl vs. TRes	0.9858
Response	Ctrl vs. TN	0.2019
Response	TnR vs. TRec	0.9759
Response	TnR vs. TRes	0.1001
Response	TnR vs. TN	0.2479
Response	TRec vs. TRes	0.4095
Response	TRec vs. TN	0.7832
Response	TRes vs. TN	0.8432

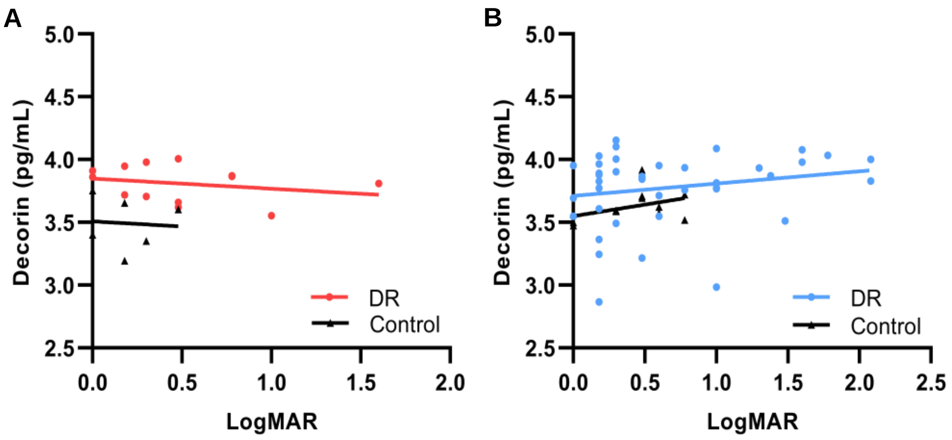

Figure S1. Correlation plot between decorin concentrations and patient visual acuity for the different sexes measured in LogMAR. Female group controls (n = 8) are represented by black triangles whilst female DR subjects (n = 13) are represented by pink circles in the scatterplot (**A**). Male group controls (n = 18) are represented by black triangles whilst female

DR subjects (n = 43) are represented by blue circles in the scatterplot (**B**). The lines represents the linear relationship between decorin concentration and LogMAR measurements. Female control (r = −0.07); female DR (r = −0.25); male control (r = 0.43); male DR (r = 0.19).
